# Impact of Red Sea Bream Iridovirus Infection on Rock Bream (*Oplegnathus fasciatus*) and Other Fish Species: A Study of Horizontal Transmission

**DOI:** 10.3390/ani13071210

**Published:** 2023-03-30

**Authors:** Kyung-Ho Kim, Gyoungsik Kang, Won-Sik Woo, Min-Young Sohn, Ha-Jeong Son, Mun-Gyeong Kwon, Jae-Ok Kim, Chan-Il Park

**Affiliations:** 1 Department of Marine Biology & Aquaculture, Institute of Marine Industry, College of Marine Science, Gyeongsang National University, 2, Tongyeonghaean-ro, Tongyeong 53064, Republic of Korea; 2 Aquatic Disease Control Division, National Fishery Products Quality Management Service, 216, Gijanghaean-ro, Gijang, Busan 46083, Republic of Korea; 3 Aquatic Disease Control Division, National Fishery Products Quality Management Service, 17, Jungnim 2-ro, Tongyeong 53019, Republic of Korea

**Keywords:** *Megalocytivirus*, histopathological, viral shedding, viral kinetics, cohabitation challenge

## Abstract

**Simple Summary:**

Red sea bream iridovirus (RSIV) has been reported to be susceptible to various marine fish species in Asian countries such as South Korea, and it causes significant economic losses due to massive mortalities in rock bream (*Oplegnathus fasciatus*) in particular. However, studies on the impact of the virus shed from RSIV-infected rock bream on other fish species are limited. Here, we investigated the dynamics of the virus by simulating the natural conditions of RSIV infection in rock bream and examining its release into seawater and the risk of horizontal transmission through cohabitation with other fish species. Our data demonstrated that the virus shed from RSIV-infected rock bream caused high mortality rates in surrounding fish species through horizontal transmission. Our findings suggest the potential for RSIV-infected rock bream in farms to spread the disease to neighboring farms. Additionally, our results demonstrated a high correlation between RSIV load and viral shedding ratio in rock breams, as well as between RSIV load and histopathological infection grades.

**Abstract:**

Red sea bream iridovirus (RSIV) causes significant economic losses in aquaculture. Here, we analyzed the pathogenicity, viral shedding, and transmission dynamics of RSIV in rock bream (*Oplegnathus fasciatus*) by employing immersion infection and cohabitation challenge models. Rock bream challenged by immersion exposure exhibited 100% mortality within 35 days post RSIV exposure, indicating that the viral shedding in seawater peaked after mortality. At 25 °C, a positive correlation between the viral loads within infected rock bream and virus shedding into the seawater was observed. Specific RSIV lesions were observed in the spleen and kidney of the infected rock bream, and the viral load in the spleen had the highest correlation with the histopathological grade. A cohabitation challenge mimicking the natural transmission conditions was performed to assess the virus transmission and determine the pathogenicity and viral load. The RSIV-infected rock breams (donors) were cohabited with uninfected rock bream, red sea bream (*Pagrus major*), and flathead grey mullet (*Mugil cephalus*) (recipients) at both 25 °C and 15 °C. In the cohabitation challenge group maintained at 15 °C, no mortality was observed across all experimental groups. However, RSIV was detected in both seawater and the recipient fish. Our results provide preliminary data for further epidemiological analyses and aid in the development of preventive measures and management of RSIVD in aquaculture.

## 1. Introduction

Red sea bream iridovirus (RSIV), the causative agent of red sea bream iridoviral disease (RSIVD), inflicts significant economic losses on the aquaculture industry [1]. Initially isolated from cultured red sea bream (*Pagrus major*) in Japan in 1990 [2], RSIV was identified in rock bream (*Oplegnathus fasciatus*) for the first time in South Korea in 1988 [3,4]. Since then, the virus has been frequently reported in several countries in East and Southeast Asia [5]. RSIV is a member of the *Megalocytivirus* genus of the family *Iridoviridae* and is reported to be highly pathogenic and infectious, with infections recorded primarily during the warm summer months [1,5]. The World Organization for Animal Health (WOAH) considers RSIVD a major threat to the health of aquatic animals and the aquaculture industry [1]. RSIV has a broad host range with over 30 species of fish known to be susceptible to infection [1,5]. Infected fishes typically exhibit blackening of the skin, anemia, splenomegaly, and abnormal swimming behavior. Histopathological examination reveals abnormally enlarged cells and necrotic lesions primarily in the spleen and kidneys [5,6,7].

RSIV-infected fishes have been shown to shed the virus into seawater, facilitating horizontal transmission through seawater, which is considered the primary route of RSIV spread [1,8]. The transmission of RSIV among fishes can occur through various routes, including waterborne transmission and direct contact with infected fish. Additionally, it was reported that the viral shed from RSIV-infected broodstock red sea bream caused a horizontal infection of juvenile red sea bream, as demonstrated by the presence of RSIV in seawater [9]. Through experimental infection under cohabiting conditions, Min et al. demonstrated that the horizontal transmission of RSIV can occur between rock bream and rockfish (*Sebastes schlegelii*) [10]. These findings suggest that the horizontal transmission of RSIV can occur between different fish species cohabiting in the same environment, highlighting the importance of studying the transmission dynamics of RSIV in multi-species aquaculture systems.

Effective disease management strategies are critical to prevent the spread of RSIV and minimize the associated economic losses. With no effective therapeutic available, the early detection of pathogens and understanding viral transmission between hosts is essential for the effective control and prevention of disease spread. The risk of the horizontal transmission of RSIV highlights the importance of developing effective disease management strategies, which should include developing sensitive and reliable diagnostic tools for the early detection of RSIV and implementing appropriate biosecurity measures to prevent the introduction and spread of the virus in aquaculture farms. Furthermore, understanding the genetic diversity of the virus and the factors that contribute to differences in susceptibility among fish species can inform the development of more effective disease control strategies. Factors such as waterborne transmission, the release of infected fish into the surrounding seawater, and the differences in RSIV susceptibility among different fish species contribute to the spread of the virus. Therefore, the risk of horizontal transmission of RSIV among fish populations poses a significant threat to the aquaculture industry.

Rock bream is known to be the most susceptible species to RSIV [1]. Out of the 244 reported cases of RSIVD in South Korea from 2013 to 2022, rock bream was the most affected species (82.8%) with 202 cases, and mortality due to RSIV occurs in rock bream every year [11]. Even though only 10 cases of RSIV were reported in red sea bream with a relatively low mortality rate [11], RSIV shed from this species can also damage surrounding farmed fish [9]. Flathead grey mullet is a major aquaculture species, representing the third largest aquaculture production in South Korea. Iridovirus was detected in farmed flathead grey mullet in Singapore between 1999 and 2003, resulting in a mortality of 10–50% [12]. In South Korea, RSIV was detected in wild flathead grey mullet in 2005 [13], and in farmed flathead grey mullet in 2014, 2017, 2021, and 2022 [11]. Flathead grey mullet is mentioned by WOAH as a fish species susceptible to RSIV; however, there is insufficient information or research on the pathogenicity of RSIV and its potential for experimental horizontal transmission to this species.

This study aimed to understand the horizontal transmission of RSIV among different fish species via sea water following viral shedding from infected rock bream. We also aimed to study the transmission dynamics and histopathological correlates of infection employing immersion infection in rock bream. Histopathological scoring and infection grade criteria were defined for RSIV-infected rock bream, red sea bream, and flathead grey mullet, based on infection kinetics. These findings provide novel insights into the risk of RSIV-infected rock bream and can help establish measures to control RSIVD in fish farms.

## 2. Materials and Methods

### 2.1. Experimental Fish

Rock bream (total length: 10.4 ± 2.7 cm; weight: 22.7 ± 7.3 g), red sea bream (total length: 9.7 ± 0.5 cm; weight: 13.7 ± 1.7 g), and flathead grey mullet (total length: 10.2 ± 0.9 cm; weight: 9.2 ± 2.7 g) were obtained from hatcheries located in Geoje and Namhae, Gyeongsangnam-do, South Korea, where RSIVD had not been reported. The fish were then acclimated in a 1600 L tank for 2 weeks. During the acclimatization period, individual tanks (1600 L) were used for each fish species, and a flow-through aquaculture system (500–1000 L/h) was employed. The system continuously supplied sand-filtered seawater passed through a 50 μm filter and treated with UV irradiation (UV-treated >30 mW/cm^2^). The water temperature was maintained at 25 ± 2 °C, and commercial feed was provided twice daily. Prior to experimentation, 15 fish from each species were randomly selected and tested for RSIV using PCR analysis, as described in the Aquatic Animal Diagnostic Test Manual for WOAH and qPCR [1,14]. Subsequent studies were conducted after confirming that the fish were not infected with the virus.

### 2.2. Virus

The RSIV strain used in this study was from a previous study [8]. To generate the crude stock of the virus, the spleen tissue of the rock bream infected with RSIV was homogenized and centrifuged at 10,000× *g* for 10 min at 4 °C. The virus supernatant was filtered through a 0.45 μm syringe filter and inoculated onto *Pagrus major* fin (PMF) cells (National Fishery Products Quality Management Service, Busan, South Korea), as described in a previous study [15], and then incubated for 7 days after RSIV inoculation. The cell culture supernatant was harvested and filtered through a 0.45 μm filter and used as an inoculum for RSIV. The RSIV copy number was determined using a quantitative polymerase chain reaction (qPCR), as described in Section 2.3.

### 2.3. qPCR and Virus Concentration in Seawater

Genomic DNA was extracted from fish tissues (25−50 mg) and blood (100 μL) using an AccuPrep^®^ genomic DNA Extraction Kit (Bioneer, Daejeon, Republic of Korea), as per the manufacturer’s manual. The extracted DNA was measured for RSIV copy number using a previously reported qPCR assay protocol [8,14]. All qPCR assays were performed using a Dice^®^ Real Time System III (Takara, Kusatsu, Japan). The RSIV concentration in seawater was determined using the flocculation method using iron chloride, as previously reported [8,16]. A total of 500 mL of seawater was collected from a fish tank and passed through a 1.6 μm pore size glass microfiber filter (GF/A; Whatman, Maidstone, UK), followed by a 0.45 μm pore MF-Millipore™ membrane filter (Merck, Darmstadt, Germany). To form Fe-RSIV flocculates using iron chloride, 4.83 g of iron (III) chloride hexahydrate (FeCl_3_∙6H_2_O) was dissolved in 100 mL of distilled water. Subsequently, 50 μL of the iron chloride solution was added to the 500 mL seawater, and the seawater containing the iron chloride solution was gently stirred at 200 rpm for 1 h at room temperature using a magnetic stirrer. The Fe-RSIV flocculates were then filtered under reduced pressure through a 0.8 μm pore size polycarbonate filter (Whatman, Maidstone, UK) fixed to a filter holder with a receiver (Nalgene, New York, NY, USA). The viruses collected on the 0.8 μm pore size polycarbonate filters were then transferred to 2 mL tubes and stored at −80 °C until DNA extraction. Filtered virus flocculates were extracted directly from the filter using the AccuPrep^®^ Genomic DNA Extraction Kit (Bioneer, Daejeon, Republic of Korea) in the same manner as previously reported [8]. The viral shedding ratio (RSIV copies/L/g) of RSIV-exposed fish was determined by dividing the average number of viral genome copies in seawater by the average weight of surviving fish in the tank. Seawater for the analysis of the viral shedding ratio was collected from the tanks where mortality occurred.

### 2.4. Histopathological Scoring and Grading of RSIV Infection

Histopathological analysis was performed on the harvested organs (brain, eye, gills, heart, kidney, liver, skin, and spleen) to score and grade RSIV infection. Each sample was fixed in 10% neutral-buffered formalin for 24 h and then refixed in the same solution for another 24 h before gradually being dehydrated with an ethanol series (70–100%). The samples were further cleared with xylene, embedded in paraffin and sectioned into 4 μm slices. Finally, the sections were stained with hematoxylin-eosin (H&E) following general protocols.

RSIV infection scoring was classified into four stages: (1) less, (2) mild, (3) moderate, and (4) severe. Each stage was scored blindly by a fish pathologist for renal and splenic lesions, both necrotic lesions and enlarged cells.

Necrotic lesions in kidneys were scored according to the following criteria: score 1 (less) if localized and present in less than 5% of the renal tubule; score 2 (mild) if present locally, but sporadically present in 5% or more of the renal tubules; score 3 (moderate) if present in parenchymal tissues and glomeruli in addition to renal tubules; score 4 (severe) when there was tubule necrosis due to necrotic lesions and atrophy due to parenchymal tissue and glomerular necrosis. Necrotic lesions of the spleen were scored based on the following criteria: focal lesions involving a small number of cells were scored as 1 (less); score 2 (mild) was given when many cells undergoing necrosis were identified; score 3 (moderate) was given if necrosis had already progressed and cell exudate and inflammatory cell infiltration had occurred; score 4 (severe) was given if necrosis had already progressed and parenchymal tissue atrophied due to necrosis.

The scoring of enlarged cells in the kidney was based on the following criteria: score 1 (less) if present locally in renal tubules and parenchymal tissues, score 2 (mild) when a small number of cells were present in renal tubules, parenchymal tissues, and glomeruli, score 3 (moderate) when 30% or more of the cells in one or more of the renal tubules, parenchymal tissues, and glomeruli were replaced by enlarged cells, and score 4 (severe) when more than 50% of the cells were replaced. Enlarged cells in the spleen were scored from 1 to 4 based on their frequency in the parenchymal tissue. The scoring standards are described in Figure S1.

Grading of the RSIV-infected samples was performed following the scoring stage and was assigned by weighting according to lesion severity. Enlarged cells representing general lesions of RSIV infection were designed to account for 70% of grading, and RSIV-induced necrotic lesions, which had a lower correlation with viral load, were designed to account for 30% of grading. Changes in other organs were recorded but not graded because of their insignificant correlation. The grading scale was as follows: Grade 0 (G0) was less than or equal to 0.2; grade 1 (G1) was greater than 0.2 and less than or equal to 0.8; grade 2 (G2) was greater than 0.8 and less than or equal to 1.5; grade 3 (G3) was greater than 1.5 and less than or equal to 2.0; grade 4 (G4) was greater than 2.0.

### 2.5. Experimental Immersion Infection of Rock Bream

The immersion infection experiment was conducted on rock bream to confirm the pathogenicity of RSIV under natural mimetic conditions. Prior to infection, each group of fish (*n* = 30) was acclimatized for two weeks in a 50 L water tank maintained at 25 °C and 15 °C. The RSIV titer in seawater was maintained at very high (10^7^ RSIV copies/mL), high (10^5^ RSIV copies/mL), medium (10^3^ RSIV copies/mL), and low (10^1^ RSIV copies/mL) doses during the 2 h 30 min immersion following virus dispensing in the rock bream tank. In this study, the RSIV challenge experiments were conducted in 16 tanks at each water temperature (25 °C or 15 °C). Eight tanks (four at 25 °C and four at 15 °C) were used to determine the cumulative mortality, and the other eight tanks (four at 25 °C and four at 15 °C) were used for time-point sampling. After immersion exposure, 100% of the seawater in all groups was replaced with fresh seawater. The control group (*n* = 30) received no treatment. Half (50%) of the rearing seawater for each group was replaced daily with seawater treated by sand filtration, 1 μm housing filter, and UV (>30 mW/cm^2^). Mortality in each group was recorded for 40 days, and viral loads were analyzed by sampling various tissues (the spleen, gills, kidneys, heart, stomach, eyes, liver, intestine, brain, skin, and muscle) of the dead fish.

To investigate the dynamics of RSIV in virus-exposed rock bream, three fish were collected at 3, 5, 7, 10, 14-, 21-, 30-, and 40 days post-exposure (dpe), and whole blood, eyes, gills, skin, livers, spleens, kidneys, hearts, and brains were collected. To measure RSIV shedding from infected fish into the rearing seawater, 500 mL (*n* = 3) of seawater was collected from a fish tank where mortality was observed, concentrated through iron flocculation, and quantitatively analyzed. For histopathological observation, tissues (*n* = 3), excluding whole blood, collected at 3, 7, 10, 14, 21, 30, and 40 days after RSIV immersion exposure in rock bream were fixed in 10% neutral formalin and analyzed as described in Section 2.4.

### 2.6. Interspecies Cohabitation Challenge

To investigate the horizontal transmission of RSIV from infected donor rock bream to naive recipient fish (rock bream, red sea bream, and flathead grey mullet), a cohabitation challenge was introduced. The donor fish groups (*n* = 30 in each group) were injected intraperitoneally (IP) with RSIV (10^6^ RSIV copies/fish), and naive rock bream, red sea bream, and flathead grey mullet (recipient groups, *n* = 30 in each group) were cohabited with the donor fish. In this study, the RSIV cohabitation challenge was conducted in 12 tanks at each water temperature (25 °C or 15 °C). Six tanks (one for each cohabitation group at 25 °C and 15 °C) were used to determine the cumulative mortality, and the other six tanks (one for each cohabitation group at 25 °C and 15 °C) were used for time-point sampling. In the cohabitation challenge experiment, donor and recipient fish were separated using cages with a mesh size that allowed for smooth water flow within the tank. To prevent the horizontal transmission of the virus shed through the fish abdomen, the donor fish’s body surface was washed with flowing seawater after virus injection, and 100% of the rearing seawater was replaced 6 h later before introducing the recipient fish. The negative control group consisted of rock bream (*n* = 30 in each group) injected with L-15 medium and naive fish (*n* = 30) cohabited. All groups of fish (donors and recipients) were maintained in 100 L tanks at 25 °C and 15 °C, and 50% of the rearing seawater was replaced daily, as previously described. The spleens and kidneys of fish (donors and recipients, *n* = 3 in each group) were sampled 1, 3, 5, 7, 10, 14, 21, 30, and 40 days after cohabitation exposure to determine the RSIV load in the fish. To investigate the load of RSIV shed from fish, 500 mL of rearing seawater (*n* = 3 in each group) was collected, concentrated, and then the virus was quantitatively analyzed. For histopathological observation, the spleen and kidney tissues (*n* = 3) collected at 3, 5, 7, 10, 14, 21, 30, and 40 days after RSIV IP injection in the fish (donors and recipients, *n* = 3 in each group) were fixed in 10% neutral formalin and analyzed as described in Section 2.4.

All experimental protocols were performed following the guidelines of the Institutional Animal Care and Use Committee of Gyeongsang National University (approval numbers: GNU-220526-E0056, GNU-220526-E0057, and GNU-220526-E0058).

### 2.7. Statistical Analysis

Statistical analyses were conducted using GraphPad Prism 9.5. An ordinary one-way analysis of variance (ANOVA) with Dunnett’s correction was performed for multiple group comparisons. Within each group, significant differences were compared to controls when the virus was initially detected. Pearson correlation coefficients were utilized to analyze the correlation between the viral shedding ratio in seawater and viral load, as well as the correlation between histopathological infection grade and viral load. Statistical significance was denoted by the following convention: * *p* < 0.05; ** *p* < 0.01; *** *p* < 0.001; **** *p* < 0.0001.

## 3. Results

### 3.1. Pathogenicity Assessment of RSIV Immersion Infection

**Figure 1:**
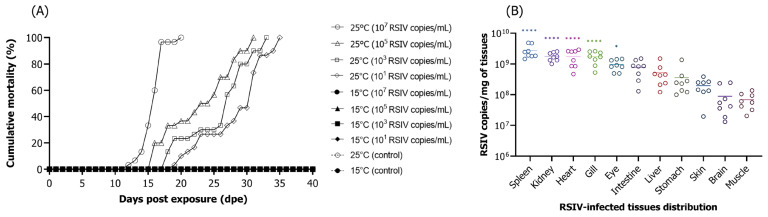
(**A**) The cumulative mortality of rock bream (*Oplegnathus fasciatus*), following immersion exposure with red sea bream iridovirus (RSIV) at 25 °C and 15 °C using four different viral concentrations (10^7^, 10^5^, 10^3^, and 10^1^ RSIV copies/mL). The control group was not subjected to any treatment. (**B**) RSIV tissue distribution in rock bream that died due to RSIV is also shown. The mean viral copy numbers (*n* = 8) are represented by bars, and asterisks are used to indicate significant differences (* *p* < 0.05; **** *p* < 0.0001) compared to the muscle.

After the immersion exposure of the rock bream with 10^7^, 10^5^, 10^3^, and 10^1^ RSIV copies/mL at 25 °C, 100% cumulative mortality was observed at 20, 31, 33, and 35 dpe, respectively (Figure 1A). At 15 °C, no mortality was observed in any immersion exposure groups nor in the negative controls (Figure 1A). The distribution of RSIV in the tissues of the fish that died (*n* = 8) following immersion exposure at 25 °C showed the highest viral load in the spleen (10^9.4^ RSIV copies/mg), which was significantly higher than the lowest viral load observed in the muscle (10^7.8^ RSIV copies/mg) (**** *p* < 0.0001) (Figure 1B).

### 3.2. Viral Load Kinetics in Rock Bream and Viral Shedding Ratio

**Figure 2:**
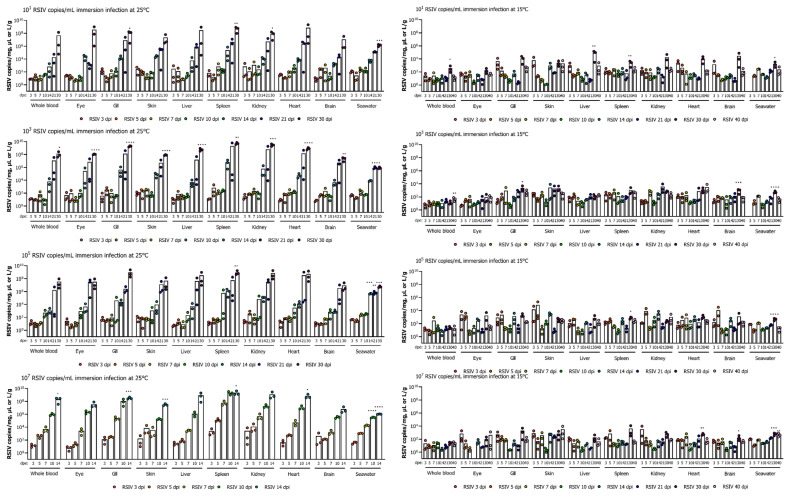
Viral load in various tissues (blood, eye, gill, skin, liver, spleen, kidney, heart, and brain) and RSIV shedding ratio in seawater after red sea bream iridovirus (RSIV) immersion exposure in rock bream (*Oplegnathus fasciatus*) at 25 °C and 15 °C at four concentrations (final concentrations: 10^7^, 10^5^, 10^3^, and 10^1^ RSIV copies/mL). The RSIV shedding ratio (RSIV copies/L/g) was determined based on the total weight (g) of the fish remaining in the tank and the number of viral copies detected in the rearing seawater. Copy number determination of RSIV was analyzed in three fish and seawater per sampling interval. Significant differences were determined using a one-way ANOVA with Dunnett’s multiple comparisons test (* *p* < 0.05; ** *p* < 0.01; *** *p* < 0.001; **** *p* < 0.0001).

In the immersion-exposed groups with 10^7^, 10^5^, 10^3^, and 10^1^ RSIV copies/mL at 25 °C, the viral loads in all tissues of rock bream tended to be relatively low (<10^4^ RSIV copies/mg) up to 10 dpe (Figure 2). After 14 dpe, the viral load rapidly increased in all tissues, with the highest viral load observed in the spleen and kidney, the major target tissues of RSIV. The group exposed to 10^7^ RSIV copies/mL tended to have a faster virus replication than other exposure concentrations. The spleen consistently had the highest viral loads at all time points sampled. RSIV was detected in seawater from 3 dpe at all concentrations of immersion exposure at 25 °C, and the amount of virus shed into seawater increased as the viral load in the fish increased. During the period of active fish mortality (12−35 dpe), the viral shedding ratio in seawater was approximately 10^4^−10^7^ RSIV copies/L/g (Figure 2).

In the RSIV immersion exposure group maintained at 15 °C, no significant differences in viral load were observed with regard to the titer of the RSIV inoculum. The virus was detected in fish until the end of the experiment, and all tissues exhibited low viral loads (<10^5^ RSIV copies/mg) until the endpoint (Figure 2). RSIV was detected in seawater at 3 dpe in the groups exposed with 10^7^ and 10^5^ RSIV copies/mL at 15 °C, and at 5 dpe in the immersion-exposed group with 10^3^ and 10^1^ RSIV copies/mL. The highest viral load in seawater was observed at 30 dpe at all concentrations of RSIV immersion exposure, which was significantly different from the first detection time point (* *p* < 0.05; *** *p* < 0.001, **** *p* < 0.0001). Viral shedding in seawater was observed up to 40 dpe, and approximately 10^1.5^ RSIV copies/L/g were shed, but no mortality was observed (Figure 2).

### 3.3. Correlation between Viral Load in Various Tissues and RSIV Shedding Ratio

**Figure 3:**
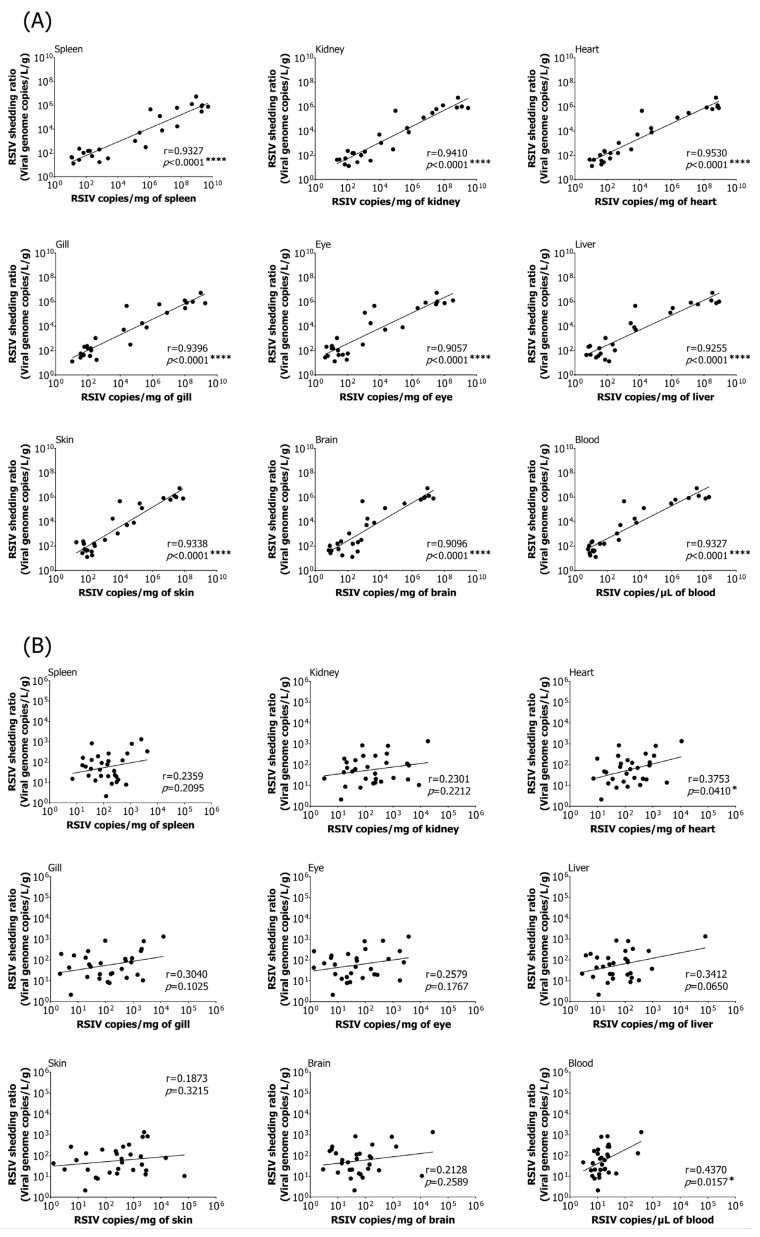
A correlation analysis was performed to examine the association between the viral load in nine different tissues (spleen, kidney, heart, gills, eyes, liver, skin, brain, and blood) and the red sea bream iridovirus (RSIV) shedding ratio in seawater after immersion exposure of rock bream (*Oplegnathus fasciatus*) with RSIV at (**A**) 25 °C and (**B**) 15 °C. The plot depicts the average viral load (*n* = 3) and viral shedding ratio (*n* = 3) in the collected fish and seawater at the same sampling time. Pearson correlation coefficients were used to determine significant differences (* *p* < 0.05; **** *p* < 0.0001).

A correlation analysis was performed to investigate the relationship between the viral load in various tissues and RSIV shedding ratios in rock bream following immersion exposure at 25 °C. Strong positive correlations were observed in all nine tissues examined, including the spleen, with the highest correlations found in the heart and kidneys (Pearson’s r > 0.90, **** *p* < 0.0001) (Figure 3A). The correlation between viral load and viral shedding ratio in the nine tissues of rock bream following immersion exposure at 15 °C was relatively low (Figure 3B). Despite the low RSIV load in fish, the virus was detected in the seawater.

### 3.4. Analysis of Histopathological Grade of RSIV Infection

The presence of histopathological lesions was confirmed by extracting the liver, skin, brain, eyes, spleen, kidney, heart, and gill tissue of RSIV immersion-exposed rock bream at 25 °C and 15 °C. Each tissue showed specific symptoms, but no significant differences were found for each RSIV immersion exposure concentration except for spleen and kidney (Figure S1).

**Figure 4:**
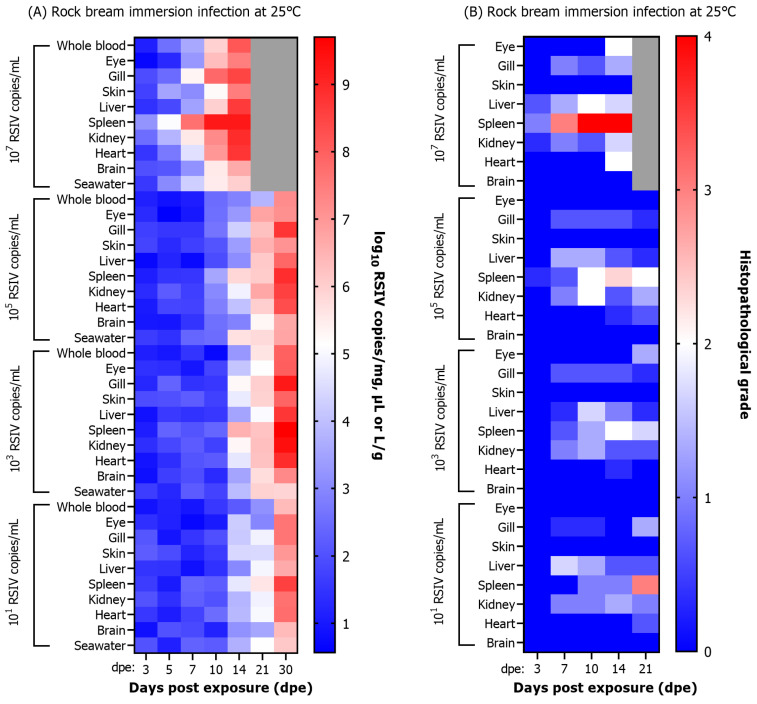
(**A**) Viral load and (**B**) histopathological grade results after immersion exposure with red sea bream iridovirus (RSIV) at four concentrations (final concentrations 10^7^, 10^5^, 10^3^, and 10^1^ RSIV copies/mL) in rock bream (*Oplegnathus fasciatus*) at 25 °C. The box represents the average of three fish, and an analysis was not performed because all fish died due to RSIV infection, as seen in the gray box (**A**,**B**).

A significant difference in the severity of RSIV-induced necrotizing lesions and enlarged cell lesions with respect to the concentrations of the inoculum was confirmed in the spleen and kidney. The viral load and histopathological infection grade were compared following RSIV immersion exposure of rock bream at 25 °C (Figure 4A,B). Changes in histopathological grades of infection were observed in the spleen among the eight tissues. A G3 lesion was observed in the spleen of a rock bream exposed with 10^7^ RSIV copies/mL at 7 dpe, with a viral load of 10^7.6^ RSIV copies/mg. G4 lesions were observed after 10 dpe, and the viral load was higher than 10^9^ RSIV copies/mg. In rock bream exposed to 10^5^, 10^3^, and 10^1^ RSIV copies/mL, G1–G3 lesions were observed in the spleen at 10–21 dpe, and the viral load was lower than 10^7^ RSIV copies/mg at this time. In conclusion, the infection grade in the spleen increased faster with higher RSIV immersion exposure concentration, and G3–G4 level lesions were confirmed at the time of fish death. The correlation between viral load and histopathological lesions was low in the kidney, the main target tissue of RSIV. Although the rock bream immersion exposed with 10^7^ RSIV copies/mL had a high intrarenal viral load of 10^8.9^ RSIV copies/mg in the kidney at 14 dpe, the histopathological infection grade was lower than G2.

**Figure 5:**
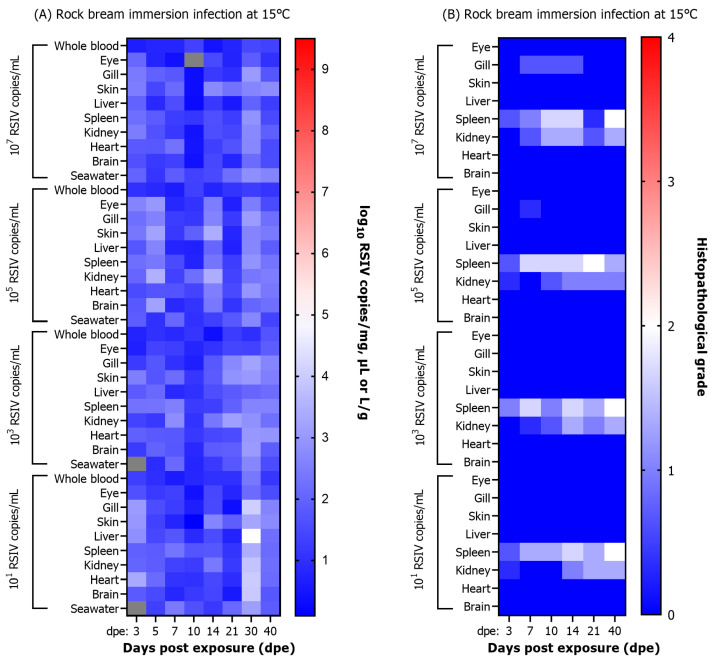
(**A**) Viral load and (**B**) histopathological grade results after immersion exposure with red sea bream iridovirus (RSIV) at four concentrations (final concentrations: 10^7^, 10^5^, 10^3^, and 10^1^ RSIV copies/mL) in rock bream (*Oplegnathus fasciatus*) at 15 °C. The box represents the average of three fish. (**A**) Gray boxes show no RSIV detected in fish and seawater.

In the RSIV immersion exposure group at 15 °C, lesions lower than G2 were observed at all exposure concentrations, and RSIVD-specific histopathological lesions were observed only in the spleen and kidney (Figure 5A,B).

### 3.5. Correlation between Viral Load and Histopathological Infection Grade

**Figure 6:**
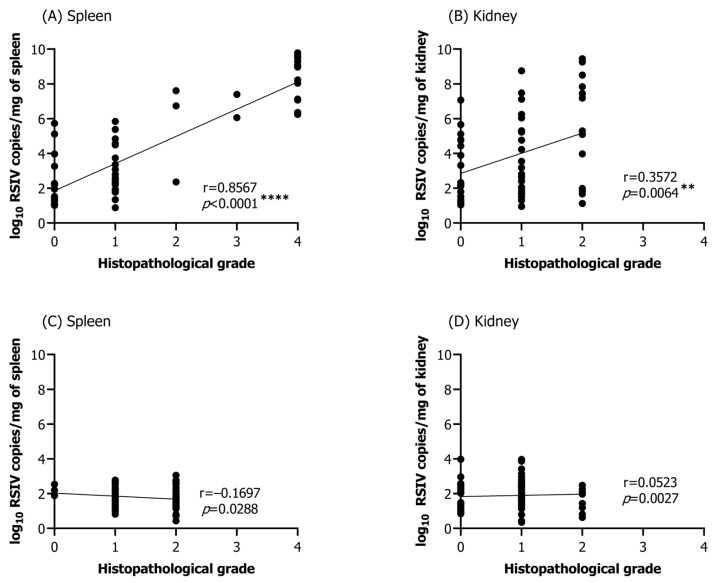
Correlation analysis between the RSIV viral load and histopathological infection grade in the spleen and kidney of rock bream (*Oplegnathus fasciatus*) infected by RSIV immersion at (**A**,**B**) 25 °C and (**C**,**D**) 15 °C. Statistical significance was determined using Pearson correlation coefficients (** *p* < 0.01; **** *p* < 0.0001).

Histopathological lesions specific to RSIVD were observed in all tissues, except for the brain and skin. However, due to the low abundance of histopathological lesions and their insignificant correlation with the viral load, the eyes, gills, liver, and heart were excluded from further analysis. Correlation analysis between viral load and infection grade was performed on selected samples in which RSIV was detected by qPCR from the spleen (25 °C group, *n* = 57; 15 °C group, *n* = 72) and kidneys (25 °C group, *n* = 57; 15 °C group, *n* = 72) of rock bream immersion exposed at 25 °C and 15 °C. In the group challenged with RSIV at 25 °C, significant correlations were found between viral load and histopathological infection grade in the spleen and kidney, the major target tissues of RSIV (spleen: Pearson’s r = 0.8567, **** *p* < 0.0001; kidney: Pearson’s r = 0.3572, ** *p* = 0.0064) (Figure 6A,B). On the other hand, in the group challenged with RSIV at 15 °C, no significant correlation was observed between the viral load and histopathological infection grades (Figure 6C,D). Histopathological infection grade (G3–G4) was exclusively observed in the spleen (Figure 6A).

### 3.6. Evaluation of Risk Associated with RSIV Cohabitation Challenge

#### 3.6.1. Interspecies Mortality

**Figure 7:**
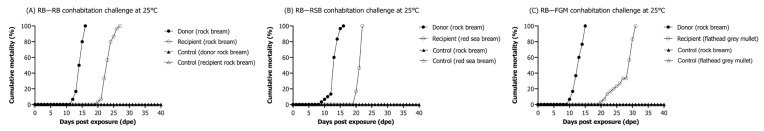
(**A**–**C**) Cumulative mortality after cohabitation challenges was evaluated in naïve rock bream (*Oplegnathus fasciatus*), red sea bream (*Pagrus major*), and flathead grey mullet (*Mugil cephalus*) recipients with donors of rock bream that were intraperitoneally (IP) injected with red sea bream iridovirus (10^6^ RSIV copies/fish) at 25 °C. The control group of donors was IP injected with 100 µL of L-15 medium (virus-free), and the recipients were not treated in any way. No mortality was observed at 15 °C.

An interspecies cohabitation challenge was introduced to assess the risk of the horizontal transmission of RSIV from infected rock bream to uninfected rock bream other and susceptible species. Mortality was assessed following cohabitation challenges between rock bream (donor) and recipient fish species, including rock bream, red sea bream, and flathead grey mullet. At 25 °C, rock bream (donor) exhibited 100% cumulative mortality at 16 dpe, while rock bream (recipient) had 100% cumulative mortality at 27 dpe (Figure 7A). Cumulative mortalities following the cohabitation challenge between rock bream (donor) and red sea bream (recipient) were 100% at 16 dpe and 22 dpe, respectively (Figure 7B). The cumulative mortalities after the cohabitation challenge between rock bream (donor) and flathead grey mullet (recipient) were 100% at 15 dpe and 31 dpe, respectively, which were slower than those observed in the challenges with rock bream and red sea bream (recipient) (Figure 7C). No deaths were observed in any groups challenged with cohabitation at 15 °C, or in the control groups at 25 °C and 15 °C (Figure 7A–C).

#### 3.6.2. RSIV Dynamics in Fish and Rearing Seawater

**Figure 8:**
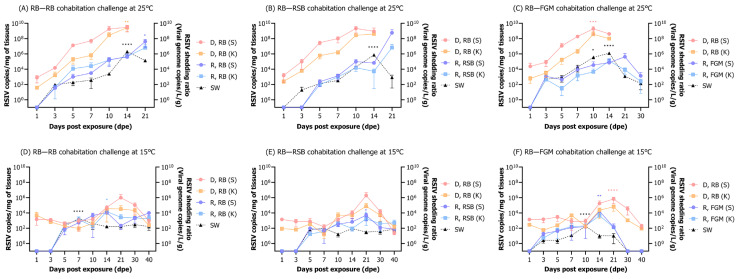
(**A**–**F**) The viral kinetics were evaluated after cohabitation challenges involving naïve rock bream (*Oplegnathus fasciatus*), red sea bream (*Pagrus major*), and flathead grey mullet (*Mugil cephalus*) (recipient, R) post red sea bream iridovirus (RSIV) intraperitoneal injection (10^6^ RSIV copies/fish) in rock bream (donor, D) at 25 °C and 15 °C. The viral load in fish was measured in the spleen (S) and kidney (K), and the virus shed from the fish into the rearing seawater was expressed as RSIV shedding ratio (SW). The RSIV shedding ratio (viral genome copies L/g) was determined based on the total weight (g) of the fish remaining in the tank and the number of viral copies detected in the rearing seawater. Copy number determination of RSIV was analyzed in three fish and seawater per sampling interval. Significant differences were determined using one-way ANOVA with Dunnett’s multiple comparisons test (* *p* < 0.05; ** *p* < 0.01; *** *p* < 0.001; **** *p* < 0.0001).

At 25 °C, rock bream (donors) were IP injected with RSIV (10^6^ RSIV copies/fish) and subsequently cohabitated with a naïve rock bream (recipients) (Figure 8A). The RSIV-exposed rock bream (donors) exhibited an increasing virus load over time, with the highest viral loads of 10^9.5^ and 10^9.4^ RSIV copies/mg observed in the spleen and kidney, respectively, at 14 dpe. The virus was detected in the rock bream (recipients) spleen and kidney at 3 dpe, with the highest viral loads of 10^7.6^ and 10^6.8^ RSIV copies/mg, respectively, observed on 21 dpe. The presence of RSIV in the seawater was detected from 3 dpe, and the shedding ratio of RSIV in seawater significantly increased during the period of observed fish mortality, which occurred between 10 and 14 dpe. At 14 dpe, the highest seawater viral load was 10^6.3^ RSIV copies/L/g, and a significant difference was observed compared to the first detection time of 3 dpe (**** *p* < 0.0001). After the complete mortality of the donor rock bream at 21 dpe, the amount of RSIV in seawater decreased to 10^5.1^ RSIV copies/L/g.

At 25 °C, rock bream (donors) were IP injected with RSIV (10^6^ RSIV copies/fish) and subsequently cohabitated with naïve red sea bream (recipients) (Figure 8B). The RSIV-exposed rock bream (donors) had the highest viral load in the spleen and kidney at 10 dpe, with 10^9.3^ and 10^8.4^ RSIV copies/mg, respectively. After cohabitation with the rock bream, the RSIV was detected in the recipient red sea bream from 5 dpe, with a sharp increase in the viral load observed in the spleen and kidney after 14 dpe. At 21 dpe, the highest viral load was observed in the spleen and kidney, with 10^8.8^ and 10^6.8^ RSIV copies/mg, respectively. The virus in seawater had the highest shedding ratio of 10^5.9^ RSIV copies/L/g at 14 dpe when rock bream (donors) mortality was active, and a significant difference was observed compared to 3 dpe, the first detection period (**** *p* < 0.0001).

At 25 °C, rock bream (donors) were IP injected with RSIV (10^6^ RSIV copies/fish), and subsequently cohabitated with naïve flathead grey mullet (recipients) (Figure 8C). The RSIV-exposed rock bream (donors) showed the highest viral load in the spleen and kidney at 10 dpe, with 10^9.3^ and 10^9.2^ RSIV copies/mg, respectively. After cohabitation with the rock bream, RSIV was first detected in the flathead grey mullet (recipients) at 3 dpe, and the highest viral load was observed in the spleen at 21 dpe, with 10^5.6^ RSIV copies/mg. The highest viral load was observed in the kidney, with 10^5.1^ RSIV copies/mg at 14 dpe. Subsequently, at 30 dpe, the viral load in the spleen and kidney decreased to 10^3.1^ and 10^2.4^ RSIV copies/mg, respectively. The highest amount of RSIV in seawater was detected at 14 dpe, with 10^6.1^ RSIV copies/L/g, showing a significant difference from 3 dpe (**** *p* < 0.0001). After that, the amount of RSIV in seawater decreased to a range of 10^2.1^–10^3.1^ RSIV copies/L/g after 21 dpe, when all rock bream (donors) had died. During this period, between 21 dpe and 30 dpe, the mortality of flathead grey mullet (recipients) was active, but the amount of virus in seawater was low.

At 15 °C, rock bream (donors) were IP injected with RSIV (10^6^ RSIV copies/fish) and subsequently cohabitated with naïve rock bream (recipients) (Figure 8D). In the spleen and kidney of the RSIV-exposed rock bream (donors), the virus load remained below 10^4^ RSIV copies/mg during 1–10 dpe. After that, the virus load increased rapidly, and at 21 dpe, the highest virus loads of 10^6^ and 10^4.5^ RSIV copies/mg were observed in the spleen and kidney, respectively. At 40 dpe, a decrease in viral load was observed in the spleen and kidney, with 10^3^ and 10^2.4^ RSIV copies/mg detected, respectively. The RSIV was first detected in the spleen and kidney of the rock bream (recipients) at 5 dpe after cohabitation with the RSIV-exposed rock bream (donors). The viral load remained below 10^5^ RSIV copies/mg throughout the entire duration of the experiment. The RSIV was first detected in the seawater at 5 dpe, and the highest shedding ratio of the virus was observed at 7 dpe, with a level of 10^3.3^ RSIV copies/L/g. Subsequently, the amount of virus in seawater steadily decreased, and on 40 dpe, it was detected at a level of 10^2.2^ RSIV copies/L/g.

At 15 °C, rock bream (donors) were IP injected with RSIV (10^6^ RSIV copies/fish) and subsequently cohabitated with naïve red sea bream (recipients) (Figure 8E). In the RSIV-exposed rock bream, the viral load in the spleen and kidney was maintained at a level of 10^4^ RSIV copies/mg during 1–10 dpe and subsequently increased to 10^6.3^ and 10^4.9^ RSIV copies/mg, respectively, at 21 dpe. At 40 dpe, the viral load had decreased to a level below 10^2^ RSIV copies/mg. The RSIV signal started appearing in the spleen and kidney of the red sea bream (recipients) at 5 dpe following cohabitation with the exposed rock bream (donors). The highest viral loads of 10^3.7^ and 10^3.2^ RSIV copies/mg were observed in the spleen and kidney, respectively, at 21 dpe, which then decreased to 10^1.9^ and 10^2.7^ RSIV copies/mg at 40 dpe. In seawater, RSIV was first detected at 5 dpe and was maintained at a level lower than 10^2^ RSIV copies/L/g until the end of the experiment.

At 15 °C, rock bream (donors) were IP injected with RSIV (10^6^ RSIV copies/fish) and subsequently cohabitated with naïve flathead grey mullet (recipients) (Figure 8F). The viral loads in the spleen and kidney of the RSIV-exposed rock bream were maintained at 10^4^ RSIV copies/mg during 1–10 dpe, followed by a sharp increase after 14 dpe. At 21 dpe, the spleen and kidney showed the highest viral load, with 10^5.8^ and 10^4.8^ RSIV copies/mg, respectively. A significant difference in the viral load was observed in the spleen compared to 1 dpe (**** *p* < 0.0001). The RSIV was first detected in the flathead grey mullet (recipients) at 3 dpe following cohabitation with an RSIV-exposed rock bream (donors), with the highest viral loads of 10^4.2^ and 10^3.6^ RSIV copies/mg detected in the spleen and kidney, respectively, at 14 dpe. There was no detection of RSIV after 30 dpe. The presence of RSIV was first observed in seawater at 3 dpe, with the highest amount of the virus, 10^2.3^ RSIV copies/L/g, confirmed at 10 dpe. No RSIV was detected in seawater after 21 dpe.

#### 3.6.3. Histopathological Infection Grade

**Figure 9:**
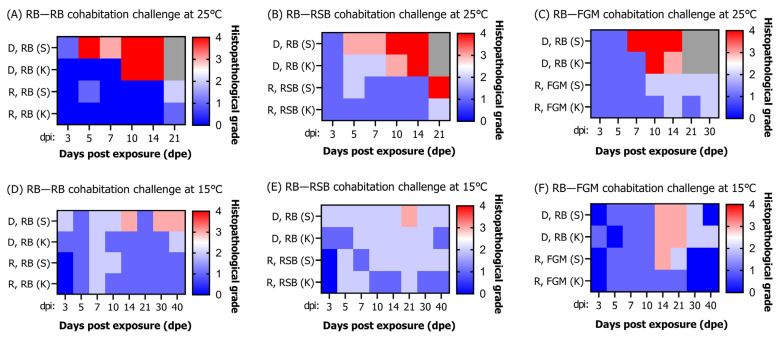
(**A**–**F**) Histopathological grade was evaluated following cohabitation challenges involving naïve rock bream (*Oplegnathus fasciatus*), red sea bream (*Pagrus major*), and flathead grey mullet (*Mugil cephalus*) (recipient, R) after intraperitoneal injection of red sea bream iridovirus (RSIV) into rock bream (donor, D) at 25 °C and 15 °C. The box indicates the average histopathological grade of the spleen (S) and kidney (K) collected from three fish. The gray box indicates that analysis could not be performed because all fish died due to RSIV infection.

In the cohabitation challenge between rock bream (donors) and rock bream (recipients) at 25 °C (Figure 9A), G1 and G0 lesions were observed in the donor spleen and kidney, respectively, at 3 dpe. At 5 dpe, G4 lesions were observed in the spleen with a high viral load of 10^7.1^ RSIV copies/mg. G4 lesions were observed in the kidney from 10 dpe, with a very high viral load of 10^8.4^ RSIV copies/mg. In the rock bream (recipients), lesions lower than G2 were observed in the spleen and kidney from 3 to 14 dpe, and the viral load was lower than 10^6^ RSIV copies/mg. At 21 dpe, when a viral load of approximately 10^7^ RSIV copies/mg was observed, G2 and G1 lesions were observed in the spleen and kidney. As a result of the cohabitation challenge between rock bream (donors) and red sea bream (recipients) at 25 °C (Figure 9B), G3 lesions were observed in the spleen and kidney of rock bream (donors) at 5–7 dpe, when a high viral load was observed. Subsequently, at 10–14 dpe, when rock bream mortality was actively occurring, approximately G4 lesions were observed in the spleen and kidney. In the red sea bream (recipients), lesions lower than G2 were observed from 3 to 14 dpe, and the viral load in the spleen and kidney was lower than 10^5^ RSIV copies/mg. Thereafter, when the viral load of 10^8.8^ RSIV copies/mg was detected in the spleen, G4 lesions were observed. In the cohabitation challenge between rock bream (donors) and flathead grey mullet (recipients) at 25 °C (Figure 9C), G1 lesions were observed in the spleen and kidney of rock bream (donors) for 3–5 dpe. Subsequently, G3–G4 lesions were observed when the viral load reached approximately 10^8^ RSIV copies/mg. In flathead grey mullet (recipients), lesions of G1–G2 were observed from 3 to 21 dpe, and the viral load was lower than 10^5^ RSIV copies/mg.

In the cohabitation challenge between rock bream (donors) and rock bream (recipients) at 15 °C (Figure 9D), G1–G2 lesions were observed in the spleen and kidney of rock bream (donors) from 3 to 10 dpe, respectively. At 14 dpe, G3 lesions were observed in the spleen with a high viral load of 10^4.7^ RSIV copies/mg. In the rock bream (recipients), lesions lower than G2 were observed in the spleen and kidney from 3 to 40 dpe, and the viral load was lower than 10^4^ RSIV copies/mg. As a result of the cohabitation challenge between rock bream (donors) and red sea bream (recipients) at 15 °C (Figure 9E), G2 lesions were observed in the spleen of rock bream (donors) from 3 to 14 dpe. Subsequently, at 21 dpe, G3 lesions were observed in the spleen with a viral load of 10^6.3^ RSIV copies/mg. In the red sea bream (recipients), lesions lower than G1–G2 were observed from 5 to 40 dpe, and the viral loads in the spleen and kidneys were lower than 10^4^ RSIV copies/mg. In the cohabitation challenge between rock bream (donors) and flathead grey mullet (recipients) at 15 °C (Figure 9F), G0–G1 lesions were observed in the spleen and kidney of rock bream (donors) from 3 to 10 dpe. G3 lesions were observed in the spleen and kidney at 14–21 dpe, with a viral load ranging from 10^4.4^ to 10^5.8^ RSIV copies/mg. Subsequently, the viral load decreased, and the histopathological infection grade decreased to G0–G2 lesions. In the flathead grey mullet (recipients), G1–G2 lesions were observed from 5 to 10 dpe, and the viral load was lower than 10^3^ RSIV copies/mg. At 14 dpe, G3 lesions were observed in the spleen with a viral load of 10^4.2^ RSIV copies/mg. After 30 dpe, no viral load was detected in the body, and no RSIV infection lesions were observed in the spleen and kidney.

## 4. Discussion

Intraperitoneal injection is generally preferred in RSIV challenge experiments owing to its high rate of infection and the lower amount of virus required. However, this route of infection is considered unnatural because the virus bypasses the host’s mucosal immune barrier, which serves as the first line of defense [17,18]. In contrast, the immersion challenge represents a more natural route of infection and is, therefore, a viable alternative for studying virus entry and the temporal progression of the disease. Although cohabitation studies offer a model that is highly similar to natural infection [19,20], it can be difficult to determine the exact infection dose and time. The primary objective of this study was to demonstrate the pathogenicity of RSIV in rock bream using an immersion challenge that mimics natural infection in fish farms. To analyze the dynamics of RSIV infection, viral shedding, and histopathological lesions in rock bream, challenge experiments were conducted at varying concentrations of the inoculum and distinct water temperatures. Additionally, a cohabitation challenge model was used to assess the risk of the horizontal transmission of RSIV.

Rock bream exposed with 10^7^, 10^5^, 10^3^, and 10^1^ RSIV copies/mL by immersion at 25 °C exhibited 100% cumulative mortality during the experimental period. In a previous study, Pacific Bluefin tuna exhibited a cumulative mortality rate of 44.9% at 22 dpe when challenged with 6.0 × 10^6^ RSIV copies/L via immersion at 25 °C [21]. However, no mortality was observed in greater amberjack when challenged with a higher exposure concentration of 3.2 × 10^7^ RSIV copies/L via immersion. To induce mortality in greater amberjack via immersion, a higher concentration (3.2 × 10^9^ RSIV copies/L) was used, resulting in a cumulative mortality rate of approximately 80% [21]. The species-specific variation in the susceptibility of fishes to RSIV may have contributed to the differences in the mortality rates. Rock bream challenged via immersion at 15 °C did not show mortality irrespective of the titer of the inoculum, which aligns with previous studies suggesting that RSIV is less virulent at lower temperatures [22]. However, when rock bream was exposed to RSIV at 15 °C, the virus was detected in the seawater as early as 3–5 dpe, with virus amounts consistently detected in seawater until the end of the 40-day experiment, despite virus amounts being lower than 10^2^ RSIV copies/L/g. This emphasizes the risk of horizontal transmission through extended periods of virus shedding. In this study, a highly significant correlation was observed between the viral load in various tissues and the RSIV shedding ratio in rock bream exposed via immersion. This finding suggests that quantification of RSIV in seawater using non-invasive methods could be useful in indirectly assessing the disease progression status of RSIV in farmed fish.

In rock bream that were immersion exposed with RSIV at 25 °C, the highest viral load was observed in the spleen among all collected organs during most stages of infection, including the early stage of infection. While the spleen and kidneys are recognized as the primary target organs for RSIV, the virus could also be detected in other organs such as the heart, gills, and eyes [1,14]. In contrast, in the group without mortality or disease progression (all challenge groups at 15 °C), a low viral load was detected in all organs examined regardless of the RSIV target organ. Previous studies have reported differences in the organ distribution of RSIV in rock bream, with asymptomatic yearling rock bream in a fish farm showing RSIV predominantly in the heart, stomach, intestines, muscles, eyes, and gills, rather than in the spleen [23]. Another study reported that the highest viral load was observed in the spleen and kidney in RSIV-infected naïve rock bream [14]. These differences suggest that yearling rock bream that survived RSIV infection may have harbored the virus without disease progression in the major target tissues, resulting in a low viral load. In previous studies, the mortality threshold for rock bream due to RSIVD infection was suggested to be >10^6^ copies/μL in the spleen [24]. In this study, while the highest viral load was observed in the spleen, fish mortality was noted after surpassing the mortality threshold, with high viral loads detected in most tissues at the time of mortality. This suggests the possibility that after the virus has sufficiently replicated in the cells of the hematopoietic tissues, which are the primary target organs in fish, the normal structure and function may collapse, leading to systemic infection in other tissues and concurrent fish mortality. In this study, the lesions of the spleen caused by RSIV infection showed a higher correlation with concentration and disease progression, while the kidney tended to have a more sensitive primary reaction (necrotic lesions at lower viral loads of RSIV). Based on these results, our study strongly suggests that the spleen is the most suitable target organ for describing the extent of the pathology in RSIV-infected rock bream.

Histopathological lesions are common in fish affected by viral infections and can be indicative of disease severity and progression [25,26]. The severity of the lesions can be influenced by viral load. Several studies have explored the correlation between viral load and histopathological lesions in fish. For example, salmonid alphavirus (SAV), a significant viral pathogen in Atlantic salmon causing severe systemic disease, exhibits a positive correlation between the severity of its associated histopathological lesions and viral load, with high levels of viral load resulting in severe histopathological changes [27]. In White sturgeon (*Acipenser transmontanus*), when challenged with *Veronaea botryosa* at 13 °C and 18 °C, histopathological lesions were observed at 13 °C, but a higher mortality rate and more severe histopathological lesion severity were observed at the more susceptible 18 °C [28]. In this study, higher mortality rates, viral loads within fish, and pronounced histopathological lesions were observed at 25 °C compared to 15 °C. These results support previous research, which suggests that the optimal temperature range for RSIV replication in vivo and in vitro is 20–25 °C, as well as observations of mortality due to RSIVD during high water temperatures in the summer at rock bream farms [29,30]. In addition to the viral load, other factors can also impact the severity of histopathological lesions in fish. Environmental factors such as temperature, water quality, and stress can all contribute to the progression of viral infections and exacerbation of the histopathological changes [31,32,33]. Histopathological analysis is a crucial tool for the diagnosis and management of viral infections in fish. The severity of histopathological lesions can help to identify the viral pathogen and determine the appropriate course of action to control the disease [34,35]. In addition, understanding the correlation between viral load and the grade of the histopathological lesions can aid in developing effective treatment strategies for viral infections in fish [36,37,38]. For example, antiviral drugs can be used to decrease the viral load and reduce the severity of histopathological changes [39,40,41]. In most studies, either quantitative analysis of the pathogen or qualitative analysis of histopathological lesions is conducted, based on the purpose of the investigation and analytical methods employed. Performing both analyses despite being challenging may yield more comprehensive results, leading to more accurate and effective diagnostic and management strategies. Consequently, it is essential to select appropriate methods of analysis depending on the research objective. In our study, we observed a significant positive correlation between viral load and the presence of enlarged cells and necrotic lesions in the spleen of rock bream infected with RSIV. Therefore, it is crucial to consider both RSIV histopathological lesions and viral load in diagnosing and managing RSIV in natural habitats and aquaculture.

The cohabitation challenge model was employed to assess the potential for horizontal transmission of RSIV from infected rock bream to uninfected rock bream, red sea bream, and flathead grey mullet as recipients. Mortality patterns and viral shedding kinetics varied based on the RSIV susceptibility of the recipient species cohabiting with the donor fish. At 25 °C, all challenge groups of rock bream (donor) exhibited a cumulative mortality rate of 100% approximately two weeks after infection, and rock bream and red sea bream (recipients) in the cohabitation challenge with rock bream (donor) died within three weeks. In contrast, flathead grey mullet showed a cumulative mortality rate of 100% at 31 dpe after the cohabitation challenge. Although the peak virus levels detected in seawater from the three cohabitation challenge groups were similar at approximately 10^6^ RSIV copies/L/g at 14 dpe, flathead grey mullet exhibited the slowest mortality rate. This suggests differences in susceptibility to RSIV among fish species and provides direct evidence that RSIV-infected rock bream can transmit the virus to other fish species horizontally. Although no mortality was observed at 15 °C, the detection of RSIV in seawater and recipient fish supports the idea that rock bream (donor) can shed the virus even at low temperatures, which may be a factor in the annual summer outbreak of RSIV. This observation is supported by a previous study where RSIV was detected in seawater at a temperature as low as 11.7 °C in a red sea bream farm [9]. However, further studies are required to investigate the duration of viral shedding following RSIV infection.

RSIV has been reported to survive in seawater at 25 °C and 15 °C for approximately 7–10 days [42]. To investigate the dynamics of RSIV in aquaculture settings, disease progression can be monitored through qPCR or histopathological analysis of fish. However, to assess the risk of disease occurrence in neighboring farms beyond the area of RSIV outbreaks, non-invasive detection of RSIV in seawater may be a valuable alternative by considering the duration of virus survival. Our study provides evidence that the virus released from rock bream, the most susceptible species to RSIV, can cause horizontal infection and mortality in other fish species through cohabitation challenges, supported by quantification of the virus in seawater. This implies that rock bream may be a significant contributing factor to the ongoing outbreaks in aquaculture.

## 5. Conclusions

Taken together, the results of this study indicate a high correlation between viral load and RSIV shedding ratio, as well as between viral load and histopathological lesions in rock bream infected with RSIV at 25 °C. Moreover, this study demonstrates the potential for the virus shed from RSIV-infected rock bream to spread the disease to other fish species.

## Data Availability

The data presented in this study are available upon request from the corresponding author.

## Supplementary Materials

The following supporting information can be downloaded at: https://www.mdpi.com/article/10.3390/ani13071210/s1, Figure S1: Scoring of lesions: Histopathological scoring of the lesions of red sea bream iridovirus-induced necrotizing lesions and enlarged cells lesions in the rock bream (*Oplegnathus fasciatus*) spleen and kidney tissues (haematoxylin and eosin stain, bar = 50 μm).
