# Supplementary material for: Impact of Red Sea Bream Iridovirus Infection on Rock Bream (Oplegnathus fasciatus) and Other Fish Species: A Study of Horizontal Transmission
Source: Animals (Basel). 2023 Mar 30;13(7):1210. doi: 10.3390/ani13071210 (PMC10093424; doi:10.3390/ani13071210)
Supplement: Supplementary file 1 [file animals-13-01210-s001.zip › Supplementary figure.pdf]

Supplementary material

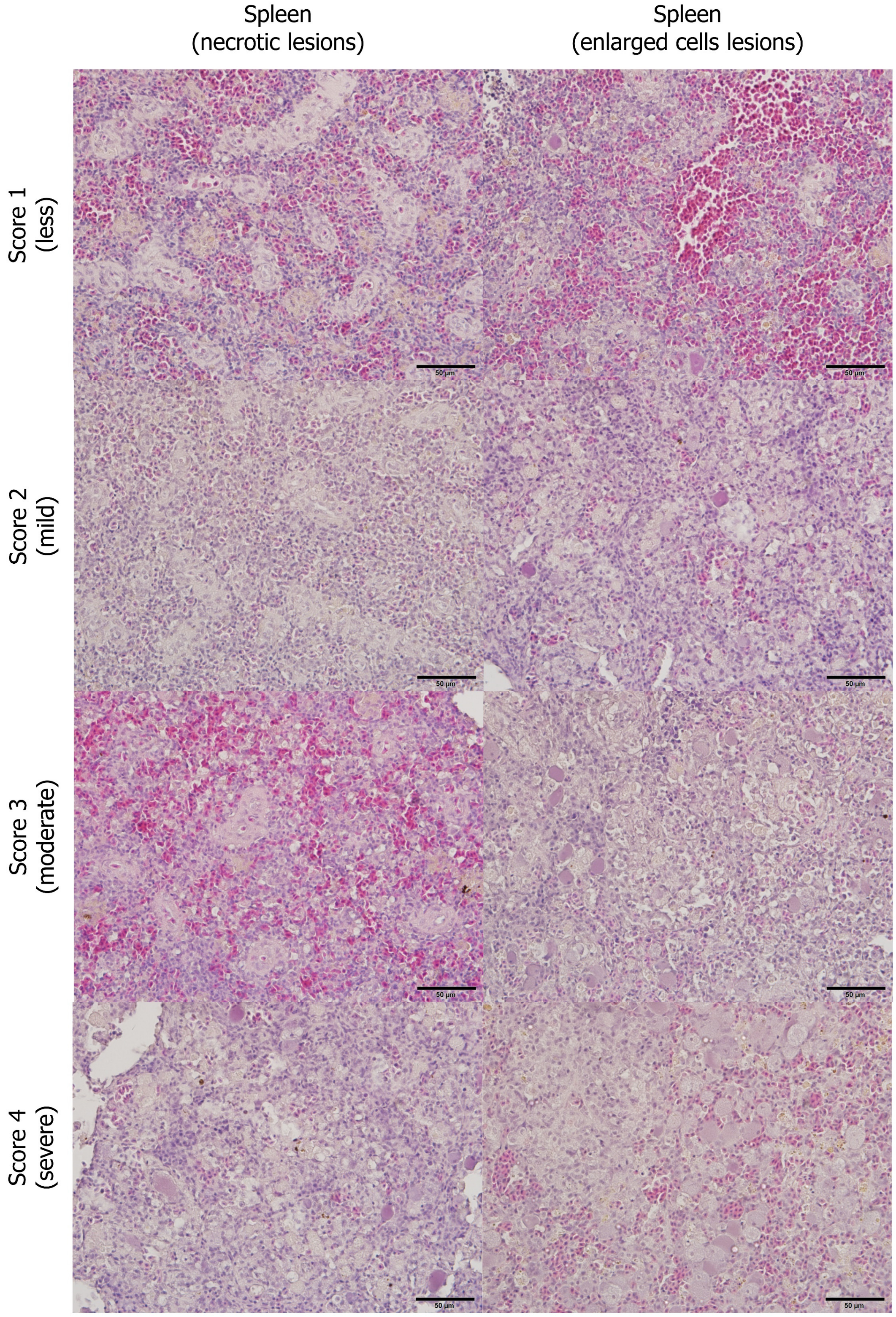

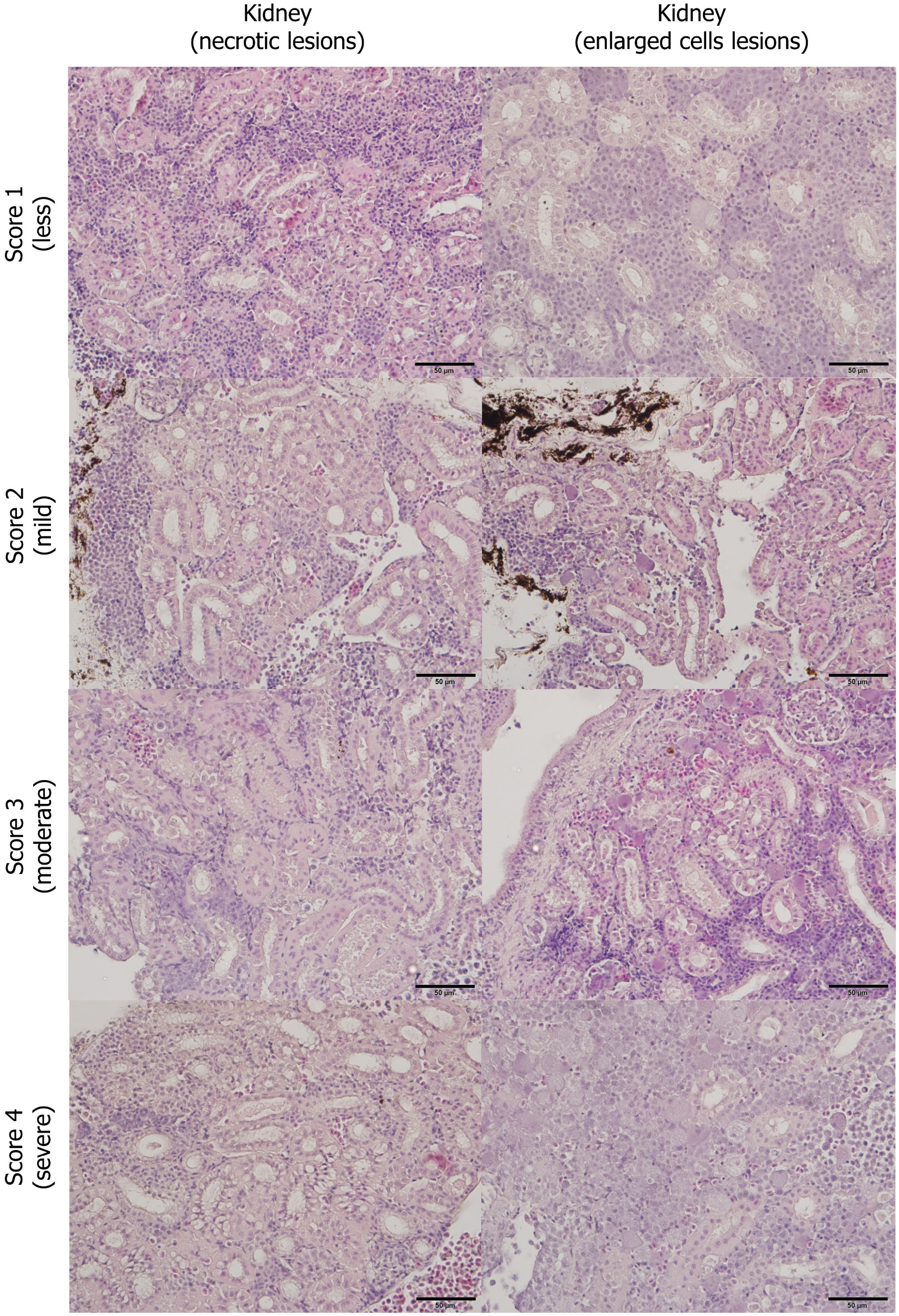
Kidney
(necrotic lesions)
Kidney
(enlarged cells lesions)
Score 1
(less)
Score 2
(mild)
Score 3
(moderate)
Score 4
(severe)
50 µm

Figure S1. Scoring of lesions: Histopathological scoring of the lesions of red sea bream iridovirus-induced necrotizing lesions and enlarged cells lesions in the rock bream (*Oplegnathus fasciatus*) spleen and kidney tissues (haematoxylin and eosin stain, bar = 50 μm).
